# Description of *Oscheius indicus* n. sp. (Rhabditidae: Nematoda) from India

**DOI:** 10.21307/jofnem-2019-004

**Published:** 2019-04-02

**Authors:** Puneet Kumar, Wajih Jamal, Vishal S. Somvanshi, Khushbu Chauhan, Sabia Mumtaz

**Affiliations:** 1 Department of Zoology, Aligarh Muslim University, Aligarh, India; 2 Division of Nematology, ICAR – Indian Agricultural Research Institute, LBS Center, PUSA Campus, New Delhi, India

**Keywords:** Amphimictic, 28S D2/D3, ITS, New species, *Oscheius*, Taxonomy

## Abstract

A new amphimictic species *Oscheius indicus* n. sp. is described and illustrated with morphological and molecular data. The species is characterized by a medium-sized and slender body (female: L = 1.1 to 1.5 mm; a = 16.8 to 20.6; b = 5.7 to 7.1; c = 7.5 to 10.4; c’ = 5.0 to 7.6; V = 45 to 51%), presence of four incisures each in the lateral fields with three minute warts, long rectum (2 to 3 anal body diameters), nine pairs of papillae arranged as 1+1+1/3+3 pattern, a prominent double-flapped epipytigma on vulval opening, presence of open leptoderan bursa and crochet needle-shaped spicules place it in the insectivora group. Morphologically, *O. indicus* n. sp. closely resembles *O. carolinensis*, *O. chongmingensis*, *O. colombiana*, and *O. nadarajani*. Molecular phylogenetic analysis carried out using ITS and D2/D3 expansion region of 28S rDNA sequences suggests that *O. indicus* n. sp. is closer to *O. chongmingensis* and *O. rugaonensis*. In summary, the morphometrical data, morphological observations and molecular phylogenetic analysis suggested that *O. indicus* n. sp. is sufficiently different from any known species and is therefore proposed as a new species within the insectivora group.

*Oscheius* is a free-living bacteriophagous or entomopathogenic nematode found in saprobic biotypes (detritus, dung), occasionally associated with beetles (Lucanidae). Körner (1954) first described *Rhabditis insectivora.* Later, Andrássy (1976) erected the genus *Oscheius* with *O. insectivorus* as its type species and considered *Oscheius* as the closest relative of *Rhabditis* (Dujardin, 1845). Sudhaus (1976) placed *Oscheius* under the family Rhabditidae and divided them into two main groups: dolichura group and insectivora group. Diagnostic characters of both the groups are unique; species under the insectivora group are characterized by leptoderan bursa, crochet needle-shaped spicules and normal rectum, whereas the dolichura group has peloderan bursa, probe head spicule tips and expandable rectum. On the basis of morphological and molecular studies, Sudhaus (2011) characterized the family Rhabditidae by generalized body plan into three groups, Pleiorhabditis, Synrhabditis, and Anarhabditis. He positioned the genus *Oscheius* under the group Synrhabditis which consisted of 27 valid species, of which 13 species belonged to dolichura group and 14 to insectivora group. Tabassum et al. (2016) recognized 28 valid species under the genus *Oscheius* of insectivora group. With the recent descriptions of *O. microvilli* (Zhou et al., 2017) and *O. safricana* (Serepa-Dlamini and Gray, 2018), at present, the number of valid species under the insectivora group of genus *Oscheius* is 30, whereas the dolichura group comprises of 14 species. Six species of the insectivora group, namely, *O. carolinensis* (Ye et al., 2010), *O. niazii* (Tabassum and Shahina, 2010), *O. siddiqii* (Tabassum and Shahina, 2010), *O. amsactae* (Ali et al., 2011), *O. microvilli* (Zhou et al., 2017) and *O. safricana* (Serepa-Dlamini and Gray, 2018) and one species in the dolichura group *O. onirici* (Torrini et al., 2015) are considered as entomopathogenic. The genus *Heterorhabditidoides* was proposed as a separate genus (Zhang et al., 2008) but it was later considered a junior synonym of *Oscheius* (Ye et al., 2010). Another species, *Heterorhabditidoides rugaoensis*, was also transferred to *Oscheius* (Darsouei et al., 2014; Tabassum et al., 2016). Synonymizing *Heterorhabditoides* with *Oscheius* has been widely accepted in the field (Liu et al., 2012; Campos-Herrera et al., 2015; Torrini et al., 2015; Tabassum et al., 2016; Valizadeh et al., 2017). A total of 44 species of *Oscheius* have been described till date, out of which 16 are from the Indian sub-continent. Here we describe the 45th species of the genus, *Oscheius indicus* n. sp.

## Materials and methods

The nematodes were extracted from soil samples collected from district Cachar, Assam, India by modified decanting and sieving and modified Baermann’s funnel techniques (Flegg, 1967). Extracted nematodes were cultured on standard NGM plates seeded with *Escherichia coli* OP50 strain as a food source as used for *Caenorhabditis elegans* cultures (Wood, 1988). Stock cultures were maintained from single gravid female. The life cycle was completed in six days at 28 °C. Nematodes were rinsed off the plates using distilled water. The nematode entomopathogenicity toward the greater wax moth, *Galleria mellonella* was evaluated by using fourth stage larvae of the greater wax moth. For light microscopy, extracted nematodes were killed and ﬁxed in hot FG (Hooper, 1970) for 24 hr and then transferred to glycerin-alcohol (Seinhorst, 1959) for slow dehydration in a desiccator. Dehydrated specimens were mounted in anhydrous glycerin on glass slides using wax ring method (De Maeseneer and d’Herde, 1963). All observations, drawing, and photographs were made on an Olympus BX 50 DIC microscope. For scanning electron microscopy, specimens were fixed in 3% glutaraldehyde in 0.05 M phosphate buffer (Kiontke et al., 2001) for 48 hr, washed in 0.05 M sodium phosphate buffer (pH = 6.9) several times, dehydrated in a graded ethanol series (30, 50, 70, 80, 90, 95, and 100%) for 20 to 25 min each, and critical-point dried in carbon dioxide. Dried specimens were mounted on stubs, coated with 20 nm gold and observed in a scanning electron microscope (Joel JSM-6510) at 15 KV.

For characterization of the ITS and 28S D2/D3 sequences for molecular taxonomy, a single nematode was taken in 25 µl of sterile water in a 0.2 ml PCR tube to which 25 µl of lysis buffer (0.2 M NaCl + 0.2 M Tris-HCl (pH 8.0) + 1% (v/v) β-mercaptoethanol + 800 µg/ml proteinase K) was added (Holterman et al., 2006). The lysis was carried out in a thermocycler at 65 °C for 3 hr with intermittent shaking followed by incubation for 5 min at 100 °C. Six µl of the lysate (diluted 1:10) was used as DNA template in PCR reaction, and the surplus lysate was stored at -20 °C. The ITS region was amplified by primers TW81 (5′-GTTTCCGTAGGTGAACCTGC-3′) (Joyce et al., 1994) and AB28 (5′-ATATGCTTAAGTTCAGCGGGT-3′) (Howlett et al., 1992); and D2 F- 5′ ACAAGTACCGTGAGGGAAAGTTG-3′ and D3 R- 5′-TCGGAAGGAACCAGCTACTA-3′ were used for D2/D3 expansion region of 28s rDNA gene (Nunn, 1992). The amplification of near full length 18s rDNA gene was attempted by primers SSU_F_07 (AAAGATTAAGCCATGCATG) and SSU_R_81 (TGATCCWKCYGCAGGTTCAC) (Gutiérrez-Gutiérrez et al., 2012). Each PCR reaction comprised of 1 unit of Platinum Taq polymerase (Invitrogen Carlsbad, CA, USA), 1x Taq polymerase buffer, 0.2 mM dNTPs, 1.5 mM MgCl_2_, 0.4 µM forward and reverse primers and 6 µl of template DNA. The thermocycling conditions for ITS were – Initial denaturation at 95 °C for 5 min, followed by 40 cycles of 95 °C for 45 sec, 55 °C for 30 sec, and 72 °C for 1.30 min, and a final elongation at 72 °C for 10 mins. For 28S D2/D3, initial denaturation was 95 °C for 5 min, followed by 40 cycles of 95 °C for 30 sec, 60 °C for 45 sec, and 72 °C for 45 sec and a final elongation of 72 °C for 10 min. The 18s rDNA was amplified as per (Gutiérrez-Gutiérrez et al., 2012); one cycle of 94 °C for 2 min, followed by 35 cycles of 94 °C for 30 s, annealing temperature of 57 °C for 45 s, 72 °C for 3 min, and finally one cycle of 72 °C for 10 min. The PCR amplified products were checked on 1% agarose gel and eluted from the gel by using Qiagen MinElute Gel extraction kit (Qiagen USA, Catalogue No. 28606). The purified products were cloned using the pGEMT-easy vector system I (Promega USA, Catalogue No. A1360), which was transformed into DH5α chemically competent cells using standard protocols. Positive colonies were screened by blue-white screening, confirmed by PCR amplification using gene-specific primers and sent for sequencing by using forward and reverse M13 primers. The forward and reverse sequences were manually edited for quality, aligned using ClustalW, and a consensus sequence was generated using BioEdit v.7.2.6 (Tom Hall, NCSU, USA). The phylogenetic analysis of the ITS and 28S D2/D3 sequences were carried out as described (Ye et al., 2018). In brief, the new and previously published sequences for each gene were aligned using ClustalW in MEGAX (Kumar et al., 2018) with default parameters. The alignments were subjected to Bayesian Inference (BI) analysis using MrBayes 3.2.6 (Ronquist et al., 2012). The General Time Reversible substitution model with gamma-distributed rate variation across sites and a proportion of invariable sites (GTR + G + I) was used as the optimal nucleotide substitution model for the analyses. A random tree was used to start the analysis for each gene and was run for 10^6^ generations and the Markov chains were sampled at intervals of 1,000 generations. Burn-in samples were discarded and other trees were used to generate a 50% majority rule consensus tree.

## Description

**Figure 1:**
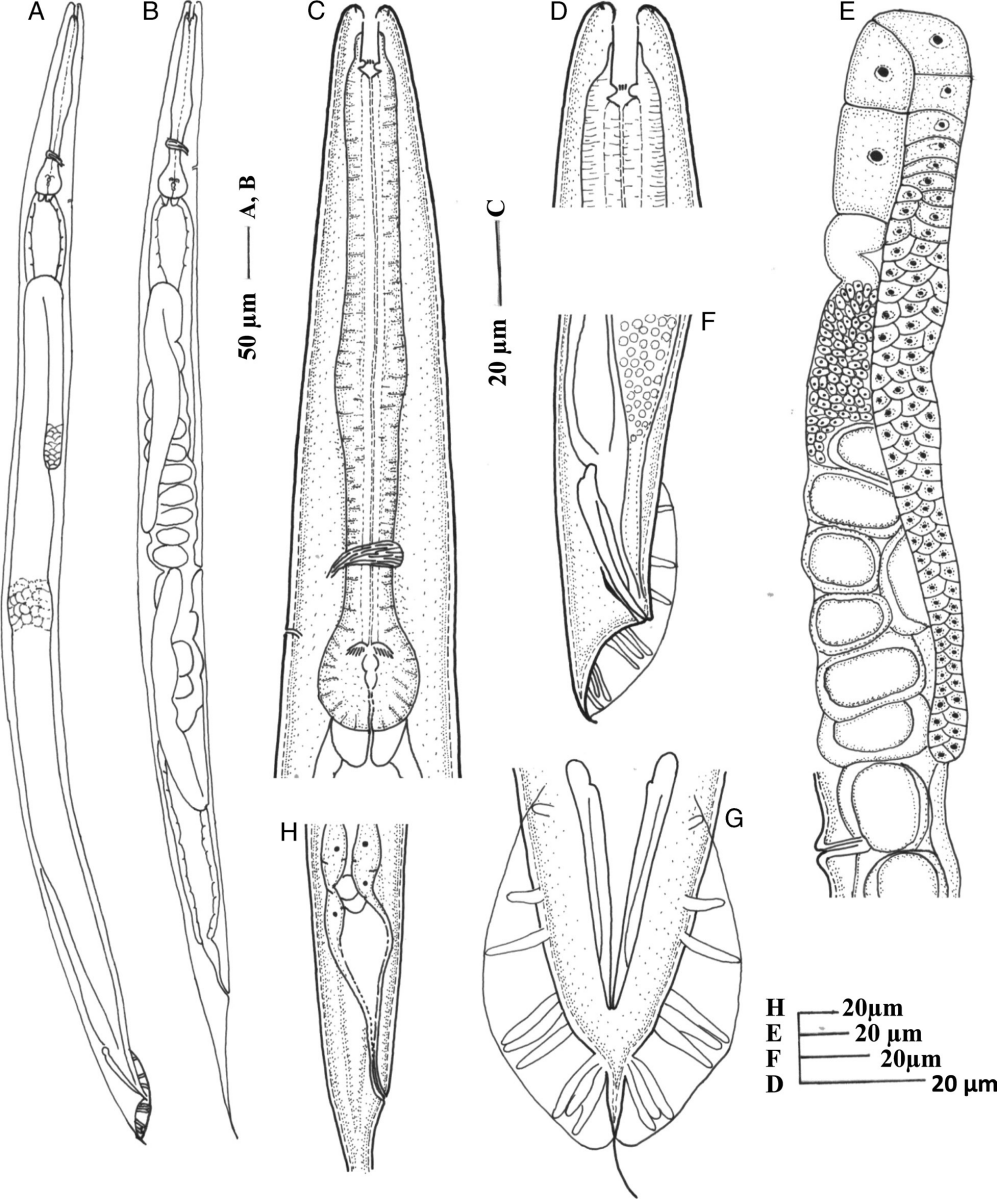
*Oscheius indicus* n. sp. (A) Entire male; (B) Entire female; (C) Pharyngeal region; (D) Anterior region; (E) Female reproductive system; (F) Male posterior region showing spicules and gubernaculum; (G) Male tail in dorso-ventral view (genital papillae and bursa); (H) Female posterior region.

*Oscheius indicus* n. sp.

(Figs 1: A–H, 2: A–I, and 3: A–D)

**Figure 2:**
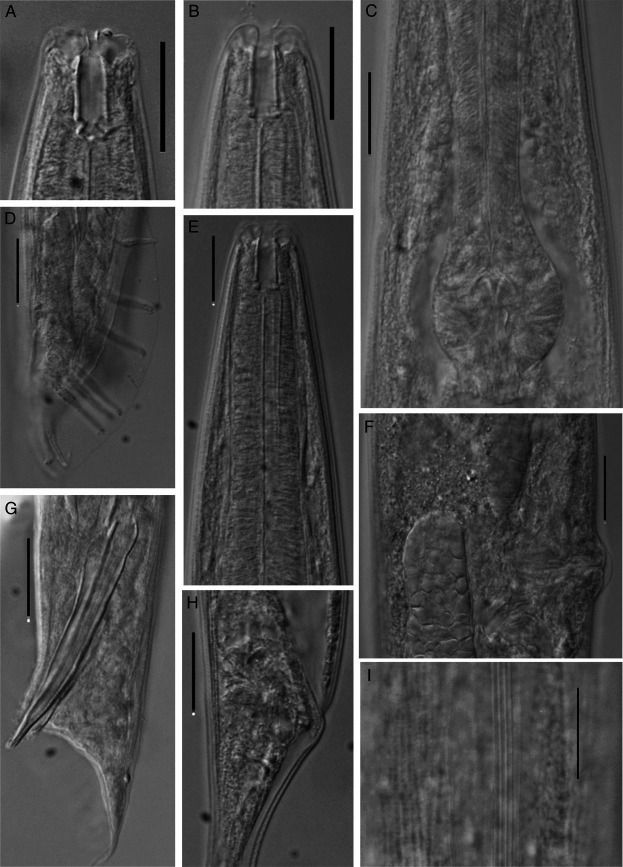
*Oscheius indicus* n. sp. (A, B) Anterior region showing stoma; (C) Posterior pharynx.; (D) Genital papillae; (E) Anterior pharynx; (F) Vulva; (G) Male posterior region; (H) Female posterior region; (I) Lateral lines. (Scale bar: 20 μm).

**Figure 3:**
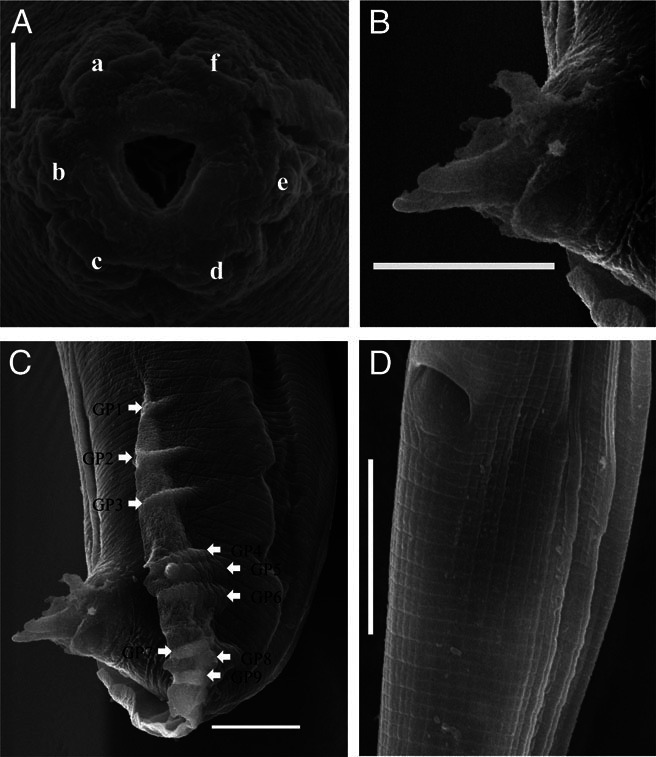
*Oscheius indicus* n. sp. (A) Enface view showing six labial papillae (a to f), (B) Crochet needle-shaped spicules (C) Male posterior region showing genital papillae (GP1 to GP9), (D) Female posterior region showing anal opening (Scale bar: A = 2 μm; B & C = 10 μm, D = 5 μm).

*Measurements*: given in Table 1.

**Table 1:** Morphometric data of *Oscheius indicus* sp. n. Measurements are in µm and in the form: mean ± standard deviation (range).

Characters	Holotype female	Paratype females	Paratype males
n	1	14	14
L	1,320	1,272.3 ± 111.7 (1,072–1,480)	1,062.7 ± 104.5 (886–1,209)
a	20.3	18.9 ± 1.1 (16.8–20.6)	19.6 ± 1.6 (17.4–22.4)
b	6.5	6.5 ± 0.4 (5.7–7.1)	6.2 ± 0.6 (4.9–6.9)
c	9.9	8.9 ± 0.9 (7.5–10.4)	31.2 ± 2.8 (26.1–36.2)
c’	5.3	5.8 ± 0.4 (5.0–6.6)	1.3 ± 0.1 (1.2–1.4)
V	50.2	48.2 ± 1.8 (45.4–50.5)	–
Maximum body diam.	65	67.8 ± 20.2 (59–82)	54.4 ± 16.3 (46–64)
Lip width	12	12.6 ± 1.1 (11–15)	11.6 ± 0.7 (11–13)
Length of stoma	16	15.4 ± 1.7 (13–18)	15.6 ± 0.9 (14–17)
Corpus	123	117.7 ± 6.1 (108–127)	105.4 ± 4.0 (101–114)
Isthmus	42	40.7 ± 3.4 (37–47)	35.4 ± 3.5 (31–41)
Basal bulb length	37	36.4 ± 2.2 (33–41)	29.9 ± 1.4 (27–32)
Basal bulb width	29	27.6 ± 1.8 (24–30)	23.8 ± 0.9 (22–25)
Pharynx	202	194.8 ± 9.8 (181–210)	172.4 ± 6.0 (159–181)
Excretory pore from anterior end	201	179.9 ± 15.1 (154–201)	173.6 ± 9.7 (158–187)
Nerve ring from anterior end	162	142.0 ± 14.1 (118–162)	136.7 ± 7.6 (122–146)
Anterior gonad	524	558.2 ± 43.2 (501–640)	–
Posterior gonad	352	504.4 ± 90.3 (324–614)	–
Vulva body diam.	64	66.4 ± 5.5 (58–80)	–
Vulva-anus distance	525	512.1 ± 40.2 (447–576)	–
Rectum	72	67.9 ± 5.8 (58–76)	54.3 ± 4.3 (46–61)
Tail length	133	142.6 ± 8.9 (131–156)	34.2 ± 2.3 (32–39)
Anal body diameter	25	24.8 ± 1.4 (23–28)	25.6 ± 1.2 (24–28)
Pharynx gonad distance	175	118.0 ± 31.6 (89–175)	82.8 ± 17.2 (61–116)
Testis	_	–	883.0 ± 82.1 (760–1,035)
Spicule length along axis	_	–	60.0 ± 4.2 (54–66)
Gubernaculum	_	–	25.1 ± 1.2 (24–28)

## Female

The female body is straight, slender, tapering toward extremities but more toward posterior end. Cuticle is finely annulated, more prominent at the mid-body region, with faint punctations all over the body. Lateral fields distinct with four lateral lines, 4–5 µm wide. Lips six, not fused, each bearing one bristle-like labial sensilla. Lip region continuous with body contour, 11 to 15 µm wide. Amphidial apertures elliptical, at the base of lateral lips. Stoma tubular, 13 to 18 µm long, 3 to 4 times longer than wide, with a thin pharyngeal collar reaching up to the 70% of stoma length. Chielostom with indistinct cheilorhabdion; metastegostmal walls usually isomorphic, with three small warts. Telostegostom narrow, funnel-shaped. Pharynx 194 µm (181–210) long; corpus cylindrical, strongly developed, 117 µm (108–127) long. Medium bulb not set off. Isthmus 41 µm (37–47) long, distinct, forming 20–22% of pharynx length. Basal bulb spherical, 36 µm (33–41) long, 28 µm (27–30) wide. Valvular apparatus prominent, haustrulum medium sized. Nerve ring located in posterior half of isthmus at 65 to 77% of pharyngeal length. Excretory pore at 85 to 95% of pharyngeal length. Deirids not observed and hemizonid obscure. Cardia 4 to 5 µm long, protruding into intestine. Phasmid conspicuous, pore like.

Reproductive system is amphidelphic. Anterior branch situated on right side and posterior branch on left side of intestine, with dorsally reflexed gonad, often extending beyond the vulva. Spermatheca consisting large sperm cells. Double-flapped epiptygma is present on the vulval opening. Uterus large, with 8 to 15 eggs. Vulva transverse slit situated at the mid-body, vulval lips protruding. Mating plug present in inseminated female. Vagina strong, thick walled about one-third to one half body width long, provided with oblique muscle. Rectum dilated, anus slit like, with protruding lips. Tail elongate, gradually tapering to fine point, 5 to 6 times as long as anal body diameter.

## Male

Male body is similar to females in general morphology, but smaller in size and with posterior region strongly curved. Testis single, reflexed ventrally on right side of intestine. Bursa leptoderan, anteriorly open, margin smooth leaving a short part of tail protruding beyond bursa. Genital papillae nine pairs, three pairs pre-cloacal, and six pairs post cloacal. Arrangement of GP1, GP2, and GP3 are either equally spaced with the length of spicule or GP1 and GP2 more spaced than GP2 and GP3. GP4 and GP5 sub-ventral and GP6 lateral in position. All are equally spaced. GP8 sub-dorsal, evenly spaced with GP7 and GP9. GP7 and GP9 sub-ventral in position. Spicules paired, separate, pointed terminus, and dagger-shaped. Gubernaculum thin, arcuate, boat shaped 42 to 44% of spicule length. Tail-1.1–1.4 anal body diameter long, protruded slightly outside the bursa.

**Figure 4:**
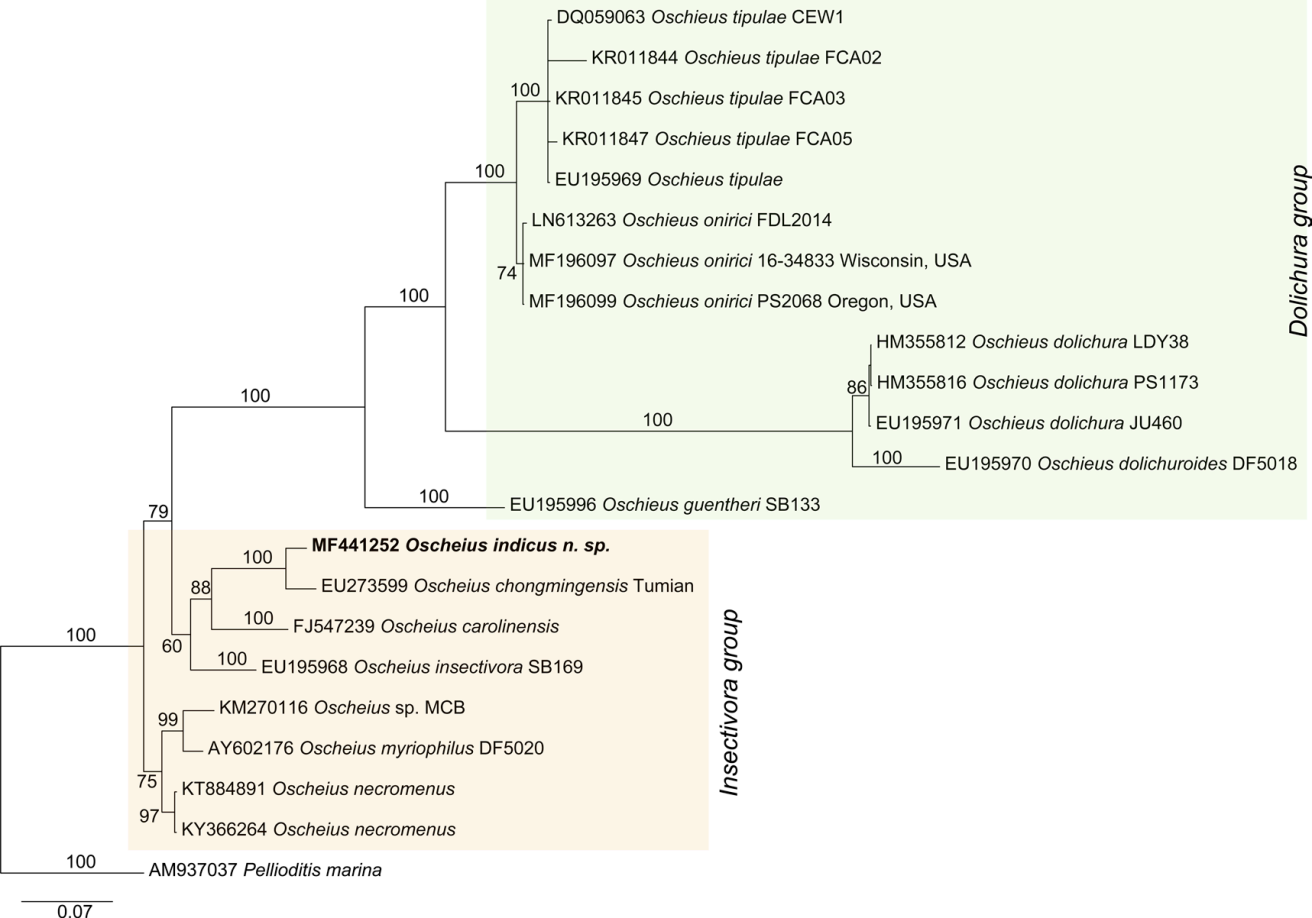
Molecular phylogenetic relationship of *Oscheius indicus* n. sp. (highlighted in bold) inferred using 28S D2/D3 extension region of 28 rDNA gene. The evolutionary history was inferred by the Bayesian analysis using the General Time Reversible substitution model with gamma-distributed rate variation across sites and a proportion of invariable sites (GTR+G+I). The posterior probability values exceeding 50% are indicated at appropriate clades. The scale bar indicates expected changes per site. Orange and green shaded boxes indicate insectivora and dolichura group, respectively.

**Figure 5:**
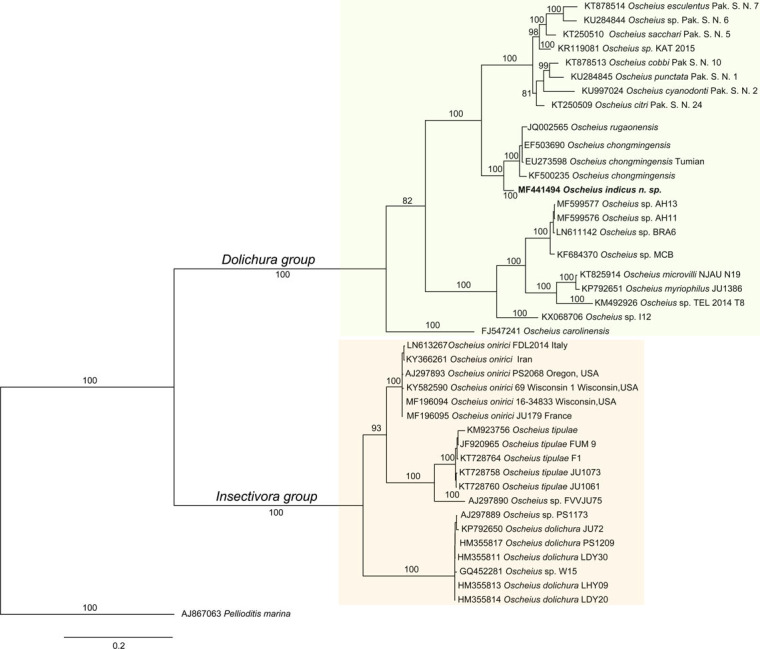
Molecular phylogenetic relationship of *Oscheius indicus* n. sp. (highlighted in bold) inferred using ITS region. The evolutionary history was inferred by the Bayesian analysis using the General Time Reversible substitution model with gamma-distributed rate variation across sites and a proportion of invariable sites (GTR+G+I). The posterior probability values exceeding 50% are indicated at appropriate clades. The scale bar indicates expected changes per site. Orange and green shaded boxes indicate insectivora and dolichura group, respectively.

The 18s rDNA sequences failed to amplify after repeated attempts. The ITS and 28S D2/D3 sequences were deposited to the GenBank with accession nos. MF441252 and MF441494. The blast search analysis of ITS identified *Oscheius* sp. (JQ002565.1) and *Oscheius chongmingensis* (EF503690, EU273598) ITS sequences as the closest matches with 92 to 91% sequence identity, 99% query coverage and E-value = 0. The blast search analysis of 28S D2/D3 sequence showed *Oscheius chongmingensis* (EU273599), *Rhabditis* sp. Hy CM-2010 (HM474859) and *Oscheius insectivora* (EU195968) as highest matching sequences at 96, 92, and 92% sequence similarity, respectively, at 93 to 100% query coverage and E-value = 0. The phylogenetic analysis using ITS and 28S D2/D3 expansion region sequences of *Oscheius indicus* sp. n. indicated that it is a member of insectivora group (Figs 4 and 5). Both the markers showed *O. chongmingensis* to be the closest relative. The ITS sequence-based tree also grouped *O. rugaonensis* along with *O. indicus* and *O. chongmingensis*. In summary, the sequences of ITS and 28S D2/D3 expansion segment of the 28s rDNA gene of *O. indicus* were sufficiently divergent from any other *Oscheius* species for which these marker sequences are available, and suggest that our species might be a new species within insectivora group (Figs 4 and 5).

## Diagnosis and Relationships

*Oscheius indicus* n. sp. is characterized by the presence of four incisures in the lateral fields, three minute warts in metastegostom, long rectum (2–3 anal body diameters), open leptoderan bursa, nine pairs of papillae arranged as 1+1+1/3+3 pattern, a prominent double-flapped epipytigma.

*Oscheius indicus* n. sp. closely resembles *O. carolinensis* (Ye et al., 2010), *O. chongmingensis* (Zhang et al., 2008), *O. colombiana* (Stock et al., 2005) and *O. nandarajani* (Ali et al., 2011).

The new species is differentiated from *Oscheius carolinensis* in having smaller stoma (13–18 µm vs 18–27 µm), shorter pharynx (181–210 µm vs 228–264 µm) and anal body diameter (24–28 µm vs 28–43 µm). In male shorter stoma (14–17 µm vs 18–23 µm), longer pharyngeal collar extension to stoma (70 vs 50%), arrangement of GP1, GP2, and GP3 (either equally or unequally spaced vs equally spaced) and GP5 (sub-ventral vs dorsal).

The new species is distinguished from *Oscheius chongmingensis* by longer stoma in female (13–18 µm vs 8.9–10.3 µm), longer tail (131–156 µm vs 67–102 µm), number of metastegostomal warts (3 vs 5), double-flapped epiptygma (present vs absent), and in males longer pharynx (181–210 µm vs 113–186 µm).

The new species can be differentiated from *Oscheius colombiana* by smaller stoma in females (13–18 µm vs 21–28 µm) and in males (14–17 µm vs 19–24 µm). Males have a shorter tail (32–39 µm vs 51–71 µm), smaller c’ ratio (1.2–1.4 vs 2.1–3.5), higher c ratio (26.1–36.2 vs 13–16), arrangement of GP5 (sub-ventral *vs* dorsal), and GP8 (sub-dorsal vs dorsal).

*Oscheius indicus* n. sp. different from *O. nadarajani* by a smaller stoma in females (13–18 µm vs 21–28 µm), shorter pharynx (181–210 µm vs 256–267 µm), longer tail (131–156 µm vs 121–123 µm) and smaller c ratio (7.5–10.4 *vs* 11–13). Males have a shorter stoma (14–17 µm vs 19.4 µm), shorter pharynx length (159–181 µm vs 243–257 µm), narrow body or smaller a ratio (17.4–22.4 vs 24–28), and in number of pre-cloacal papillae (3 pairs vs 4 pairs).

Due to the presence of leptoderan bursa, crochet needle-shaped spicules and ITS and 28S D2/D3 molecular marker sequences showing sufficient difference to the closely related genera, we propose the position of the new species under insectivora group. The nematode did not show any entomopathogenicity against *Galleria mellonella*. Any invertebrate associate for this species is not known.

## Type habitat and locality

Collected from soil samples of fallow fields from district Cachar, Assam, India.

## Type specimens

Holotype female on slide *Oscheius indicus* n. sp. /1, and 10 paratype females and 10 paratype males on slides *Oscheius indicus* n. sp. /2 to 10 are deposited in the nematode collection of Department of Zoology, Aligarh Muslim University, Aligarh, India and 4 paratypes females and 4 paratype males slides of *Oscheius indicus* n. sp. /11 to 14 are deposited in the National Nematode Collection of India housed at the Division of Nematology, ICAR – Indian Agriculture Research Institute, New Delhi, India.

## Discussion

The genus *Oscheius* has a large number of species which are morphologically very close to each other. Sudhaus (2011) divided the species into two groups – dolichura group and insectivora group. Species under dolichura grou*p* are represented by species with slender body, peloderan bursa, inconspicuous posterior phasmids and spicules with straight tip (without a distal hook), whereas species under insectivora group are characterized by large and wide body with 4 to 6 incisures each in the lateral fields with 3 to 5 ridges, pseudopeloderan/leptoderan bursa, phasmids posterior to the last genital papillae and crochet-needlelike spicules (with a distal hook). Variations have been observed in the arrangement of papillae within the same species. In some specimen of *O. indicus*, GP1, GP2, and GP3 are evenly spaced, while other show an uneven distribution with GP1 which is spaced at greater distance as compared to GP2. We compared it to other species that shows similarity with respect to the distribution of the papillae. *O. carolinensis* have evenly distributed pre-cloacal papillae while *O. chongmingensis*, *O. colombiana*, and *O. nadarajani* have unequal arrangements with GP1 more spaced than GP2 and GP3. Presence of three minute warts in *O. indicus* n. sp makes it different from *O. chongmingensis* (3/5 warts), *O. shamimi* (2 warts), and many species that have varying numbers of warts.

The phylogenetic analysis using ITS and 28S D2/D3 expansion region sequences revealed the phylogenetic position of the proposed new nematode species in the insectivora group. The molecular finding is further supported by the morphological characters like the presence of leptoderan bursa and crochet needle-shaped (with a distal hook) spicules. Therefore, based on ITS and 28S D2/D3 molecular marker sequences as well as morphological characteristics which are distinctly different from those of the closely related species, the present species is being reported as a new species under insectivora group.
